# A Double-Blind Study on Acupuncture Sensations with Japanese Style of Acupuncture: Comparison between Penetrating and Placebo Needles

**DOI:** 10.1155/2018/8128147

**Published:** 2018-04-24

**Authors:** Masako Nishiwaki, Miho Takayama, Hiroyoshi Yajima, Morihiro Nasu, Joel Park, Jian Kong, Nobuari Takakura

**Affiliations:** ^1^Department of Acupuncture and Moxibustion, Faculty of Health Sciences, Tokyo Ariake University of Medical and Health Sciences, 2-9-1 Ariake, Koto-ku, Tokyo 135-0063, Japan; ^2^Department of Physiology, Showa University School of Medicine, 1-5-8 Hatanodai, Shinagawa-ku, Tokyo, Japan; ^3^Department of Psychiatry, Massachusetts General Hospital, Harvard Medical School, Charlestown, MA 02129, USA

## Abstract

To investigate the acupuncture sensations elicited by the Japanese style of acupuncture, penetrating acupuncture and skin-touch placebo needles were randomly administered at various insertion depths (5 and 10 mm for the penetrating needles and 1 and 2 mm for the placebo needles) at LI4 to 50 healthy subjects. Among the 12 acupuncture sensations in the Massachusetts General Hospital Acupuncture Sensation Scale (MASS), “heaviness” was the strongest and most frequently reported sensation with the 10 mm needles, but not with the 5 mm needles. There were no significant differences in number of sensations elicited, MASS index, range of spreading, and intensity of needle pain for 5 mm penetration versus 1 mm skin press and 10 mm penetration versus 2 mm skin press. The MASS index with 2 mm skin-touch needles was significantly larger than that with 1 mm skin-touch and 5 mm penetrating needles. The factor structures in the 12 acupuncture sensations between penetrating and skin-touch needles were different. The acupuncture sensations obtained in this study under satisfactorily performed double-blind (practitioner–patient) conditions suggest that a slight difference in insertion depth and skin press causes significant differences in quantity and quality of acupuncture sensations.

## 1. Introduction

De qi, a characteristic constellation of sensations felt by patients during acupuncture needling, has long been regarded as an important factor related to clinical effects [1]. For the last several decades, much effort has been made to investigate the quantity and quality of acupuncture sensations in detail. Acupuncture sensation questionnaires such as the Acupuncture Sensation Scale (ASS) [2], the Subjective ASS (SASS) [3], the Massachusetts General Hospital ASS (MASS) [1], and the Southampton Needle Sensation Questionnaire (SNSQ) [4] have been developed as tools to objectively scrutinise acupuncture sensations. Using these questionnaires, acupuncture sensations elicited by manual acupuncture, electroacupuncture [3, 5], and various other manipulations [6] have been studied. Also, comparisons of acupuncture sensations among acupoints [2, 3, 7] and between acupuncture-experienced and acupuncture-inexperienced subjects [8, 9] have been made. The Standards for Reporting Interventions in Clinical Trials of Acupuncture currently recommend reporting de qi in clinical studies [10, 11].

Acupuncture sensations are an essential factor in inducing an analgesic effect [3] and improving local blood flow in the skin and muscles [12, 13]. The relationship between acupuncture sensations and analgesic effects has been clearly demonstrated in clinical trials of acupuncture treatment on epicondylalgia [14] and osteoarthritis of the knee [15, 16]. In recent randomised controlled trials, acupuncture sensation questionnaires were utilised to examine the relationship between acupuncture sensations and efficacy of acupuncture treatment [3, 17–25]. In some of these studies, acupuncture sensations reported on the MASS with placebo/sham acupuncture needles were analysed [17, 22–25] in order to better understand acupuncture-specific sensations, as well as the mechanism of acupuncture treatment.

However, there have been several limitations to these studies. First, the studies were single-blinded, with the practitioner being aware of which needle was being applied. Second, practitioners did not keep the insertion depth, and in the case of placebo needles, skin press, constant throughout the trials. Also, the methods of acupuncture used in most of the above studies were of the Chinese style, limiting their validity in other styles, such as the Japanese style of acupuncture. Almost all of the studies that investigated acupuncture sensations were performed using 0.2–0.38 mm diameter needles [2, 3, 6, 7, 9, 12–15, 19–21], which are relatively large diameter needles not usually used in Japanese acupuncture. Unlike Chinese acupuncture, Japanese acupuncture typically uses thin needles, 0.16–0.18 mm in diameter [26], with a shallow insertion of <5 mm in depth [27] and a tapping-in method to penetrate the skin using a guide tube [28]. Although there have been clinical studies that employed the Japanese style of acupuncture to investigate de qi, patients reported only whether they felt de qi, as well as a dull sensation, and a skin-penetration sensation [29–32]. To the best of our knowledge, no report has used acupuncture sensation questionnaires to analyse acupuncture sensations with the Japanese style of acupuncture.

In this study, we investigated acupuncture sensations elicited with needles in the Japanese style of acupuncture. The study was double-blinded (practitioner–patient) and insertion depth and skin press were kept constant. To measure the sensations, we translated the MASS into Japanese (Japanese MASS) and reported criterion-related validity, construct validity, and reliability [33]. At present, MASS is the most plausible candidate for a standard tool since it is the most frequently applied scale in clinical studies in English-speaking areas. Further, it has already been translated into Chinese, and this version has been validated for clinical use [34]. The aim of this study was to investigate and compare acupuncture sensations elicited with penetrating needles inserted 5 and 10 mm deep and with nonpenetrating, skin-touch placebo needles pressed 1 and 2 mm deep.

## 2. Methods

### 2.1. Study Design and Participants

The study design was a randomised, double-blinded (practitioner–patient), and crossover clinical trial comprising four conditions. Overall, 50 healthy subjects (32 males, 18 females, aged 22.3 ± 7.5 years) who had normal Japanese language ability and no nervous system disorders were recruited. All subjects had acupuncture experience and knowledge about acupuncture, including acupuncture sensations or de qi. One acupuncturist with 12 years of acupuncture treatment experience (male, aged 35 years) applied the treatment. The acupuncture treatment was performed in a laboratory at Tokyo Ariake University, Tokyo, Japan. The laboratory was maintained at 24°C–26°C.

The study was approved by the Ethics Committee of the Tokyo Ariake University of Medical and Health Sciences (approval number 198). The study objectives and protocol were explained to each participant using a written form, and all participants provided written consent.

### 2.2. Interventions

**Figure 1:**
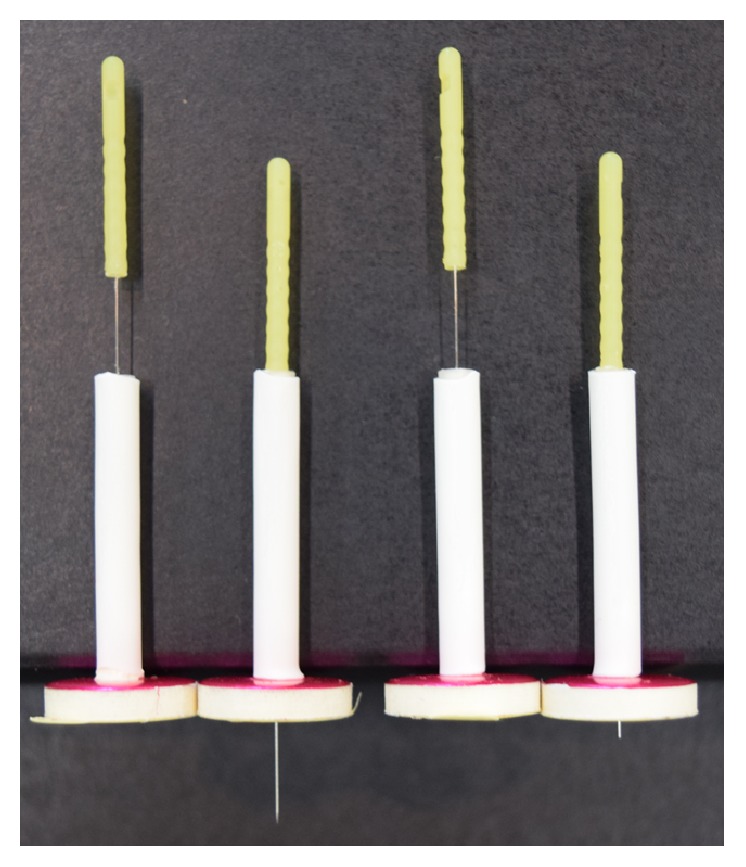
*Takakura needles for double blinding before and after insertion*. Penetrating needle inserted 5 or 10 mm deep (left) and skin-touch placebo needle pressed into the skin 1 or 2 mm deep (right).

We used penetrating acupuncture needles and skin-touch placebo needles designed for double-blinding (practitioner–patient) [28–31, 35–37] (Figure 1). For each subject, we prepared 5 mm penetrating needles to insert 5 mm from the body surface, 10 mm penetrating needles to insert 10 mm from the body surface, 1 mm skin-touch needles to press into the skin 1 mm with a protruding blunt tip, and 2 mm skin-touch needles to press into the skin 2 mm with a protruding blunt tip. The appearances of the four needles were indistinguishable. The diameter of the needles was 0.18 mm.

Based on our previous study using ordinary acupuncture needles [33], the needles were applied at LI4 (large intestine meridian) [38], the most frequently used point in acupuncture research [2, 3, 7–9, 22, 32, 33], on the right or left hand with a rotating technique until the needle handle made contact with the top of the opaque tube, which was followed by the tapping-in method [28]. After needle insertion, the acupuncturist rotated the needle by 180 degrees clockwise and anticlockwise alternately at 1 Hz for 30 s [5, 8, 9, 39, 40] and then removed it immediately.

### 2.3. Procedure

Fifty sets of four needles (a 5 mm penetrating needle, a 10 mm penetrating needle, a 1 mm skin-touch needle, and a 2 mm skin-touch needle) were sealed in bags for sterilisation, and then they were sterilised. Before applying the needles, the needle tips were set just above the bottom of a guide tube for penetrating needles and just above the lower stuffing for skin-touch placebo needles.

Before the trial, all 50 subjects and the acupuncturist were informed that penetrating and/or skin-touch needles would be used.

The subjects lay on their backs with their arms resting along their sides. The acupuncturist disinfected each subject's skin between the first and second metacarpal bones on the back of both hands with ethanol. Then he picked a needle randomly from the set of four and applied the needle at LI4. After the first needle, one of the three remaining needles was applied at LI4 on the other hand. In the second trial after a one-week interval, he applied the remaining two needles at LI4 on both hands in the same way as in the first trial. To exclude bias, we randomly assigned subjects to right or left LI4 for the first needling using a web-generated table of random numbers (http://randomization.com). In the second trial, right and left LI4 were applied in the reverse order to the first trial. As a result, the four types of the needles were applied to both hands in random order (chi-square test, *p* = 0.27).

Immediately after removal of each needle, subjects completed the following questionnaires: (1) the Japanese MASS, on which subjects rated the intensity of each acupuncture sensation corresponding to the 12 descriptors on a numerical rating scale (NRS) from 0 (none) to 10 (strongest imaginable); if the subjects felt other sensations besides the 12 descriptors, they reported the additional sensation and rated it on the NRS; (2) a human figure to shade the range of spreading of acupuncture sensation to be evaluated as 0 (none), 1 (localized), 2 (digit/wrist), 3 (lower forearm), 4 (upper forearm), and 5 (beyond) according to the MGH Acupuncture Sensation Spreading Scale [1]; and (3) the visual analogue scale (VAS) to evaluate needle pain, on which subjects rated the intensity of pain from 0 (no pain) to 100 (most severe pain, unbearable).

To evaluate the degree of subject and practitioner blinding, the subjects and acupuncturist were asked to record whether they thought the needle was “penetrating,” “skin-touch,” or “unidentifiable.” They then rated their confidence in their guess on a 100 mm VAS for confidence from 0 (no confidence) to 100 (complete confidence) [28, 30, 36, 37].

The acupuncturist and subjects also reported adverse events during each needling, if any.

### 2.4. Statistical Analysis

The Friedman test and Dunn's multiple comparison test among and between groups were conducted for the numbers and intensities of the 12 descriptors in the MASS, MASS indices (the magnitude of overall acupuncture sensations) [1], range of spreading, intensities of needle pain, and subjects' confidence. Frequencies of the 12 descriptors were compared among and between the four types of needles with Cochran's *Q* test and Dunn's multiple comparison test. For subjects' guesses, Kappa coefficients for 5 mm penetrating needles versus 1 and 2 mm skin-touch needles and for 10 mm penetrating needles versus 1 and 2 mm skin-touch needles were calculated. Further, Bang's blinding index (BI) for each type of needles was calculated by the numbers of guesses. The Mann–Whitney *U* test was used to identify differences in intensities for the 12 MASS descriptors and degrees of spreading between subjects who guessed a needle they received as “penetrating” and subjects who guessed a needle they received as “skin-touch.”

To evaluate factor structures of acupuncture sensations, we performed an exploratory factor analysis according to the Japanese MASS ratings with penetrating and skin-touch needles. Furthermore, to estimate which acupuncture sensations in the MASS were related to subjects' guesses at needle authenticity, we added “penetrating” in subjects' guesses as a variable and performed an exploratory factor analysis. A principal component extraction method was used in the factor analyses. Factors with eigenvalues greater than 1.0 were extracted and a varimax rotation involving Kaiser normalisation below 25 iterations for convergence was performed.

Statistical analyses were performed using SPSS Version 24 (IBM Japan, Ltd., Tokyo, Japan).

## 3. Results

### 3.1. Numbers of Acupuncture Sensations Elicited, MASS Index, Spreading of Acupuncture Sensation, and Intensity of Needle Pain with Each Type of Needles

The upper rows in Table 1 show the numbers of acupuncture sensations elicited with each type of needles, the MASS index, range of spreading, and the intensity of needle pain. The upper rows in Table 2 show the comparisons among the four needles for each index. There were no significant differences in the four indices between 5 mm penetration and 1 mm skin press and between 10 mm penetration and 2 mm skin press.

**Figure 2:**
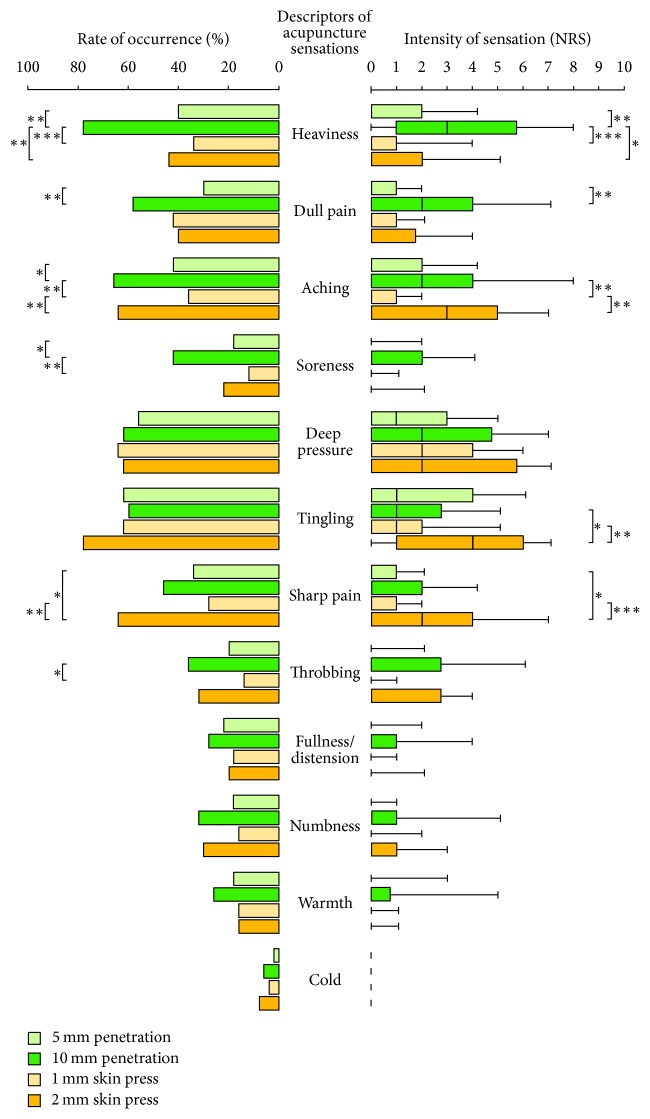
*Occurrence frequency and the intensity of acupuncture sensations on the Japanese MASS (n = 50).* The top, middle, and bottom lines of the boxes correspond to the 75th, 50th, and 25th percentiles, respectively. The whiskers extend from the 90th percentile to the 10th percentile. ^*∗*^*p* < 0.05, ^*∗∗*^*p* < 0.01, and ^*∗∗∗*^*p* < 0.001.

The rates of occurrence and intensities of each of the 12 acupuncture sensations are shown on the left and right in Figure 2, respectively. More than 50% of subjects felt “deep pressure” and “tingling” with all four types of needles.

“Heaviness” was statistically characterised by both occurrence frequency and intensity with 10 mm penetration, but not with 5 mm penetration, 1 mm skin press, or 2 mm skin press. The 2 mm skin-touch needles tended to induce greater “tingling,” “sharp pain,” and “aching” compared with the 1 mm needles.

### 3.2. Relationship between Subjects Guess and Intensity of Acupuncture Sensations

The intensities of “heaviness” and “aching” with penetrating needles guessed as “penetrating” were significantly higher than those with penetrating needles guessed as “skin-touch” (“heaviness”: *p* = 0.001, “aching”: *p* = 0.015). As for skin-touch needles, the intensities of “heaviness,” “aching,” “dull pain,” and “soreness” for needles guessed as “penetrating” were significantly higher than those guessed as “skin-touch” (“heaviness”: *p* = 0.002, “aching”: *p* = 0.015, “dull pain”: *p* = 0.027, and “soreness”: *p* = 0.030). No significant difference in the intensity of “sharp pain” was revealed between correct and incorrect guesses by subjects (penetrating needles: *p* = 0.087, skin-touch needles: *p* = 0.310).

### 3.3. Factor Analysis

Tables 3 and 4 show the results of factor analysis in the 12 acupuncture sensations with penetrating and skin-touch needles, respectively. Factors 1, 2, and 3 of penetrating needles were inferred as “deep dull-heavy sensation,” “no pain sensation,” and “superficial pain sensation.” Factors 1, 2, and 3 for skin-touch needles were inferred as “superficial pain sensation,” “deep dull-heavy sensation,” and “thermal sensation.” Factor 4 of skin-touch needles, which was a prominent difference from penetrating needles, was inferred as “soreness.”

To explore the association between MASS sensations and subjects' guesses at needle authenticity, we added “penetrating” in subjects' guesses as a variable and performed an exploratory factor analysis.  Table 5 shows the results of factor analysis that include “penetrating” in subjects' guesses as an additional variable in the 12 acupuncture sensations. “Penetrating” in subjects' guesses was categorised as the third factor with “heaviness,” “aching,” and “dull pain.”

### 3.4. Blinding Assessments of Needles

The lower rows in Tables 1 and 2 show BIs for subjects' and practitioner's guesses, confidence in guesses, and the Kappa coefficients between needles applied (penetrating or skin-touch needles) and subjects' or practitioner's guesses (penetrating or skin-touch).

For the practitioner, the BIs of each type of needles were close to zero and his confidences were very low, which indicates that his guesses were random.

For the subjects, although the blinding statuses of the 10 mm penetrating needles and the 1 mm skin-touch needles were assessed as “unblinded,” there were no significant differences among the four types of needles in their confidences and no significant difference in subjects' confidence between correct and incorrect guesses for both penetrating (*p* = 0.155) and skin-touch (*p* = 0.819) needles, which indicates that the subjects could not identify the nature of the needle. Except for subject blinding for 10 mm penetrating needles versus 1 mm skin-touch needles, the Kappa coefficients indicated that the degree of agreement was poor for both the subjects and the practitioner.

### 3.5. Adverse Events

Dot haemorrhage occurred in three cases with the 10 mm penetrating needle. There were no critical adverse events.

## 4. Discussion

In this study, we applied double-blind 5 and 10 mm penetrating needles and 1 and 2 mm skin-touch needles to investigate acupuncture sensations with the Japanese style of needling using Japanese MASS. We found that “heaviness” was a distinctive feature for 10 mm needle penetration. The total number of sensations elicited, the MASS index, range of spreading, and the intensity of needle pain with the 5 and 10 mm penetrating needles were similar to those with the 1 and 2 mm skin-touch needles, respectively. However, the factor structures of acupuncture sensations were different between penetrating and skin-touch placebo needles, implying that acupuncture penetration and insertion depth may be associated with different sensation patterns.

Literature suggests that the depth of needle insertion, that is, shallow (minimal/superficial) or deep insertion, affects acupuncture sensation [41–44] and efficacy [45–48]. This indicates that the depth of needle insertion is an influential factor in determining the sensation and efficacy of acupuncture and that insertion depth should be controlled when investigating acupuncture sensations. In this study, we applied Takakura needles for double blinding and for maintaining a fixed insertion/touch depth [35]. The needles for double blinding can blind patients and practitioners to exclude any bias from psychological factors. Previous studies suggested that acupuncture sensations can be elicited by phantom acupuncture or sham laser acupuncture, which implies that patient-practitioner interaction may alter acupuncture sensations [49, 50]. Therefore, we believe that the acupuncture sensations obtained in this study were unbiased in terms of technique.

“Heaviness” was revealed as a distinctive sensation with 10 mm penetration insertion (Figure 2). The results were similar to those of our previous study using Japanese MASS, in which 10 mm penetration with ordinary needles of 0.18 mm diameter was applied [33]. Literature suggests that “heaviness,” “aching,” “dull pain,” and “soreness” are all regarded as important acupuncture sensations with manual acupuncture [3, 7, 17, 51]. We found that the sensations of “aching,” “dull pain,” and “soreness” from 2 mm skin-touch needles were not different in frequency and/or intensity from 10 mm penetrating needles (Figure 2). Furthermore, although spreading of acupuncture sensations has been considered as one of the specific characteristics of de qi [18, 52–54], the spreading with 2 mm skin-touch needles was similar to that with 10 mm penetrating needles (Table 1). Also, despite the finding that stimulating the fascia and muscles was reported to elicit “dull,” “heavy,” and “spreading” sensations frequently [43], our results indicated that only “heaviness” was exclusive to deep needle insertion. Similar occurrences of “deep pressure” and “tingling” with the four types of needles meant that these sensations were also not exclusive to 10 mm penetrating needles.

With 5 mm penetrating needles, “tingling” and “deep pressure” were as frequently reported as with 1 and 2 mm skin-touch needles, and “heaviness” was much less elicited compared with 10 mm penetration (Figure 2). Despite needle insertion, “heaviness” was not a specific sensation with 5 mm penetration. Acupuncture sensations with 5 mm penetrating needles were similar to those with 1 and 2 mm skin-touch needles, which endorses the potential feasibility of blinding patients using these needles.

The differences in acupuncture sensation between 10 mm penetration (deep insertion) and 5 mm penetration (shallow insertion) indicate that a slight difference in insertion depth should not be ignored when acupuncture sensations are evaluated. The majority of indications for acupuncture sensations with 10 mm penetration were higher than those with 5 mm penetration in this study, suggesting that acupuncture sensations with shallow insertions tend to be weaker than those with deep insertions [41, 42, 44]. If the needle grasp felt by acupuncturists as de qi is due to mechanical coupling of the needle with connective tissue [55], a needle depth of 10 mm should contact larger connective tissues than that of 5 mm. This may explain why the 5 mm penetration did not elicit “heaviness,” which was exclusive to deep insertion, if the de qi felt by acupuncturists is assumed to be simultaneously felt by patients.

The tip of the 2 mm skin-touch needle is estimated to reach the subcutaneous tissues according to an MRI study performed at LI4 [56]. The stimulation of such superficial tissues with a 2 mm skin press may produce strong sensations. The 2 mm skin-touch needles could be a reliable placebo control for 10 mm penetrating needles because of the similarities in number of sensations elicited (except for “heaviness”), MASS index, range of spreading, and intensity of needle pain between these needles.

Interestingly, we found that acupuncture sensations elicited with 1 mm skin-touch needles were quite similar to those with 5 mm penetrating needles as determined by similarities in number of sensations elicited, MASS index, range of spreading, intensity of needle pain, and the occurrence and intensity of every descriptor of the acupuncture sensations. These results suggest that 1 mm skin-touch needles could be a reliable placebo control for 5 mm penetrating needles.

We found that the intensity of acupuncture sensations was entirely different, especially aching, tingling, and sharp pain, between 1 and 2 mm skin-touch needles. This may provide insight into some inconsistent results in other studies using other superficial placebo needles. Several results on acupuncture sensations with single-blind skin-touch needles have been reported. For instance, White et al. investigated acupuncture sensations in patients suffering from hip or knee pain between Streitberger placebo needles and penetrating acupuncture needles and concluded that the Streitberger placebo needle is a convincing control based on similarities in intensity for “dull,” “radiating,” “stinging,” and “electric” sensations [20]. Liang et al. reported that pain scores with sham needle applications using the Park device were larger than those with real penetrating needles [40]. In contrast, it was reported that the intensities of “aching” with verum needles, which is considered to be equivalent to pain [17, 22], were higher than those with the Streitberger placebo needles. Further, it was reported that the intensities of acupuncture sensations with single-blind placebo needles were considerably dependent on needling by practitioners [20, 57]. Taken together, these studies call for a better way to control the depth/pressure of the nonpenetrating placebo needle in acupuncture research.

Using factor analyses of the 12 acupuncture sensations on the Japanese MASS, the construct validity of MASS was confirmed using double-blinded, controlled-depth needles. Considering that all subjects had experienced acupuncture in addition to the double blinding of this study, an extraction of the three factors latent in the 12 descriptors, including a factor of sensations unrelated to pain such as “warmth,” “numbness,” and “fullness/distension,” was more valid and reliable than the extraction of the two factors revealed in our previous, unblinded study, in which half of the subjects had not experienced acupuncture. Furthermore, this is the first factor analysis of acupuncture sensations with skin-touch placebo needles under practitioner blinding. The factor structure of acupuncture sensations with real penetrating needles was different from that with skin-touch placebo needles. For instance, although “soreness,” which was categorised as a “dull-heavy” sensation, was reported to be one of the particular sensations experienced with real penetrating needles [17], the soreness elicited with skin-touch placebo needles in this study was not involved in the “dull-heavy” factor.

It is worth noting that patient blinding was almost successfully conducted from the Kappa coefficients and subjects' confidence. Also, successful blinding for the practitioner was confirmed with 53.5% unidentified needles in addition to the Kappa coefficients and blinding indices. These results indicate that the methods used in this study have removed much of the bias present in previous studies.

Consistent with previous observations that “sharp pain” is frequently elicited by acupuncture stimulation in the epidermis, dermis, and shallow subcutaneous tissue [43], “sharp pain” was the most predominant sensation with 2 mm skin-touch needles in this study. In a previous study, for subjects who believed that the phantom acupuncture they received was real acupuncture, the intensities of “dull pain” and “heaviness” were similar to those with real needles, but those of “sharp pain” and “tingling” were less than those with real needles [49]. In the current study, however, no significant difference in the intensity of “sharp pain” was revealed between correct and incorrect guesses by subjects. “Sharp pain” was not a distinguishing cue for the subjects. To be more precise, the decisive cues for subjects for identifying the needles as “penetrating” seemed to be “aching,” “dull pain,” and “heaviness” from the factor analysis, including subjects' guesses as additional variables.

This was the first study to investigate acupuncture sensations with penetrating needles and skin-touch placebo needles while keeping the depth of insertion and skin press constant. The results obtained in this study suggest that a slight difference in the depth of needle insertion and skin press significantly influences the quantity and quality of acupuncture sensations, which indicates a future direction of studies on acupuncture sensations. Double-blind needles [35] must play a significant role in future clinical acupuncture trials.

There are several limitations in this study. Only one acupuncturist participated in this study. We thus cannot test interpractitioner credibility in this experiment. Also, we only recruited healthy subjects. Acupuncture sensations in patients are reported to be different from healthy subjects [53, 58]. Future studies are needed to evaluate the sensations of different types of needles in patient populations.

## 5. Conclusion

Potential factors in the 12 acupuncture sensations on the Japanese MASS were different between double-blinded penetrating and skin-touch placebo needles. The strongest and most frequent sensation observed was “heaviness” for 10 mm penetration. The total number of sensations elicited, the MASS index, range of spreading, and the intensity of needle pain with 1 and 2 mm skin-touch needles were similar to those with 5 and 10 mm penetrating needles, respectively. A slight difference in the depth of insertion and skin press caused significant differences in the intensity and quality of acupuncture sensations.

## Figures and Tables

**Table 1:** Indices of acupuncture sensations and patient and practitioner blinding for each type of needle.

	Penetrating needles		Skin-touch needles	
	5 mm penetration	10 mm penetration	1 mm skin press	2 mm skin press
*Indices of acupuncture sensations*				
Number of acupuncture sensations elicited	3.8 ± 3.2	5.5 ± 3.3	3.6 ± 2.8	4.9 ± 2.7
MASS index	2.2 (2.6 ± 2.3)	3.6 (4.1 ± 2.6)	1.6 (2.5 ± 2.0)	4.4 (4.4 ± 2.2)
Spreading range	0 (0.6 ± 1.1)	2 (1.3 ± 1.2)	0 (0.4 ± 0.9)	0 (0.7 ± 1.2)
Intensity of needle pain	1.6 (13.5 ± 21.2)	11.5 (24.7 ± 28.7)	0 (8.5 ± 16.9)	24.5 (31.2 ± 29.8)
*Blinding assessment*				
Patient blinding				
Confidence in guess	61.6 (61.3 ± 25.1)	70.7 (65.1 ± 27.8)	71.9 (65.8 ± 26.8)	76.8 (70.2 ± 26.2)
Blinding index (95% CI)	0.12 (−0.14 to 0.38)	0.52 (0.29 to 0.75)	0.30 (0.04 to 0.56)	−0.08 (−0.35 to 0.19)
[Blinding status]	[Random guess]	[Unblinded]	[Unblinded]	[Random guess]
Practitioner blinding				
Confidence in guess	5.5 (15.8 ± 24.2)	11.0 (30.8 ± 35.9)	6.9 (6.6 ± 2.8)	10.0 (18.9 ± 27.6)
Blinding index (95% CI)	0.02 (−0.16 to 0.20)	0.18 (−0.02 to 0.38)	−0.32 (−0.49 to −0.15)	−0.14 (−0.32 to 0.04)
[Blinding status]	[Random guess]	[Random guess]	[Random guess]	[Random guess]

*Note*. MASS index, ranges of spreading, intensity of needle pain, and confidence level in needle guesses on 100 mm VAS are expressed in median (mean ± SD); confidences in subjects' and practitioner's guesses were calculated without unidentified needles. Unidentified needles (% of each type of 50 needles) by the subjects were 12% for 5 mm penetration, 4% for 10 mm penetration, 2% for 1 mm skin press, and 4% for 2 mm skin press. Unidentified needles (% of each type of 50 needles) by the practitioner were 58% for 5 mm penetration, 46% for 10 mm penetration, 52% for 1 mm skin press, and 58% for 2 mm skin press.

**Table 2:** Comparisons among the four types of needles for determining indices of acupuncture sensations and patient and practitioner blinding.

	Comparisons between 4 types of needles	Comparisons between penetrating and skin-touch needles				Comparisons between depths	
		5 mm penetration versus 1 mm skin press	5 mm penetration versus 2 mm skin press	10 mm penetration versus 1 mm skin press	10 mm penetration versus 2 mm skin press	5 mm versus 10 mm penetration	1 mm versus 2 mm skin press
*Indices of acupuncture sensations*							
Number of acupuncture sensations elicited	^*∗∗*^ *p* = 0.001	*p* = 1.00	*p* = 0.134	^*∗∗*^ *p* = 0.006	*p* = 1.00	^*∗*^ *p* = 0.036	^*∗*^ *p* = 0.028
MASS index	^*∗∗∗*^ *p* < 0.001	*p* = 1.00	^*∗∗∗*^ *p* < 0.001	^*∗∗*^ *p* = 0.001	*p* = 1.00	^*∗∗∗*^ *p* < 0.001	^*∗∗∗*^ *p* < 0.001
Spreading range	^*∗∗∗*^ *p*< 0.001	*p* = 1.00	*p* = 1.00	^*∗*^ *p* = 0.012	*p* = 0.199	^*∗*^ *p* = 0.045	*p* = 1.00
Intensity of needle pain	^*∗∗∗*^ *p*< 0.001	*p* = 1.00	^*∗*^ *p* = 0.017	^*∗∗∗*^ *p* < 0.001	*p* = 1.00	^*∗*^ *p* = 0.036	^*∗∗∗*^ *p* < 0.001
*Blinding assessment*							
Patient blinding							
Confidence in guess	*p* = 0.277	—	—	—	—	—	—
*κ* coefficient	—	0.22	0.03	0.42	0.23	—	—
[Strength of agreement]		[Poor]	[Poor]	[Moderate]	[Poor]		
Practitioner blinding							
Confidence in guess	^*∗*^ *p*= 0.040	*p* = 1.00	*p* = 0.701	*p* = 0.096	*p* = 1.00	*p* = 0.228	*p* = 0.375
*κ* coefficient	—	−0.30	−0.14	−0.17	0.00	—	—
[Strength of agreement]		[Poor]	[Poor]	[Poor]	[Poor]		

*Note*. ^*∗∗∗*^*p* < 0.001, ^*∗∗*^*p* < 0.01, and ^*∗*^*p* < 0.05; confidences in subjects' and practitioner's guesses were calculated without unidentified needles.

**Table 3:** Factor loadings on the Japanese MASS for penetrating needles (*n* = 100).

Variables	Factor		
	1	2	3
Heaviness	0.821		
Deep pressure	0.752		
Aching	0.732		0.522
Soreness	0.711		
Dull pain	0.702		
Sharp pain	0.592		0.513
Throbbing	0.542		0.489
Warmth		0.786	
Fullness/distension		0.722	
Numbness		0.653	0.512
Tingling			0.791
Cold		0.510	0.676

*Note*. Factor loadings over 0.45 are exclusively represented. Cumulative contribution = 0.707. *N* = 100 indicates 50 of each of 5 mm and 10 mm penetrating needles.

**Table 4:** Factor loadings on the Japanese MASS for skin-touch needles (*n* = 100).

Variables	Factor			
	1	2	3	4
Tingling	0.842			
Sharp pain	0.829			
Numbness	0.711			
Aching	0.698			
Fullness/distension	0.482			
Dull pain		0.803		
Heaviness		0.800		
Deep pressure		0.672		
Cold			0.648	
Throbbing	0.525		0.594	
Soreness				0.776
Warmth			0.549	0.617

*Note*. Factor loadings over 0.45 are exclusively represented. Cumulative contribution = 0.668. *N* = 100 indicates 50 of each of 1 mm and 2 mm skin-touch needles.

**Table 5:** Factor loadings of the Japanese MASS including “penetrating” in subjects' guesses for all needles as an additional variable (*n* = 189).

Variables	Factor		
	1	2	3
Fullness/distension	0.730		
Warmth	0.701		
Soreness	0.659		
Dull pain	0.604		0.432
Deep pressure	0.571		
Tingling		0.861	
Sharp pain		0.723	
Aching	0.409	0.572	0.479
Numbness	0.544	0.564	
Throbbing	0.499	0.520	
Cold		0.494	
“Penetrating” in subjects' guesses			0.819
Heaviness	0.555		0.653

*Note*. Factor loadings over 0.45 are exclusively represented. Cumulative contribution = 0.594. *N* = 189 indicates number of needles excluding those guessed as “unidentifiable.”
